# Esophageal tuberculosis induced dysphagia: a case report

**DOI:** 10.1186/s12876-022-02211-2

**Published:** 2022-03-22

**Authors:** Dylan Olson, Kevin C. Liu, Anthony P. Merza, Ermias Tilahun, A. Aziz Aadam

**Affiliations:** 1 Department of Medicine, Northwestern University, Feinberg School of Medicine, 251 E Huron St, Chicago, IL 60611 USA; 2 Division of Gastroenterology and Hepatology, University of Arizona College of Medicine, Tucson, AZ USA; 3 Methodist Hospital Chicago, 5025 N Paulina St, Chicago, IL 60640 USA; 4 Division of Gastroenterology and Hepatology, Northwestern University, Feinberg School of Medicine, 251 E Huron St, Chicago, IL 60611 USA

**Keywords:** Esophageal tuberculosis, *Mycobacterium tuberculosis*, Esophageal tuberculosis induced dysphagia

## Abstract

**Background:**

Patients can present for a wide variety of etiologies for dysphagia, and it is important to consider less common causes once common etiologies have been ruled out. Extrapulmonary *Mycobacterium tuberculosis* (TB) presentations are rare to see in the western populations due to relative lack of TB exposure and overall less immunocompromised populations, but should be considered for at-risk patients. Gastrointestinal (GI) TB is rare, and the GI tract is considered only the sixth most frequent site of extrapulmonary TB (EPTB).

**Case presentation:**

This is a case report of a 35-year-old Ethiopian male presenting with dysphagia and retrosternal odynophagia who was found to have infiltration of mediastinal lymphadenopathy into the esophageal wall secondary to TB. This patient underwent an upper endoscopy, which revealed a linear 2 cm full thickness mucosal defect in the middle esophagus concerning for an infiltrative process with full thickness tear. Computed tomography (CT) of the chest demonstrated a subcarinal soft tissue mass that was inseparable from the esophagus. He was referred to thoracic surgery and underwent an exploratory mediastinal dissection. A mediastinoscopy scope was inserted and the mediastinal dissection was made until the subcarinal nodes were identified and removed. Biopsy results showed necrotizing and non-necrotizing granulomas, and acid-fast bacilli (AFB) culture from the surgically removed lymph node showed *Mycobacterium TB* complex growth. He had no known TB exposures and did not have any TB risk factors. He then followed up in infectious disease clinic and was managed with anti-tuberculosis treatment (ATT) with complete resolution of symptoms.

**Conclusions:**

Our patient was ultimately found to have esophageal TB secondary to mediastinal invasion into the esophageal wall from lymphadenopathy associated with TB. This is an extremely rare presentation in western populations due to diminished exposure rates and overall less immunocompromised populations compared to impoverished countries with increased TB exposure and human immunodeficiency virus (HIV) infection rates. Although TB is not as commonly seen in western populations, it should be considered on the differential for any atypical presentations of GI diseases for patients with clinical or geographic risk factors.

## Background

*Mycobacterium tuberculosis* infection affects nearly 10 million people a year and causes 1.5 million deaths [1]. This number is significantly lower in the United States as seen in 2019, at 2.7 cases per 100,000 [2]. TB is common in the immunosuppressed population with 12% of all new diagnoses occurring in human immune deficiency virus (HIV) positive patients. Extrapulmonary TB (EPTB) occurs in 12% of patients with active TB infection of which 3.5% is hepatobiliary and 6–38% is intra-abdominal [3].

## Case presentation

**Fig. 1:**
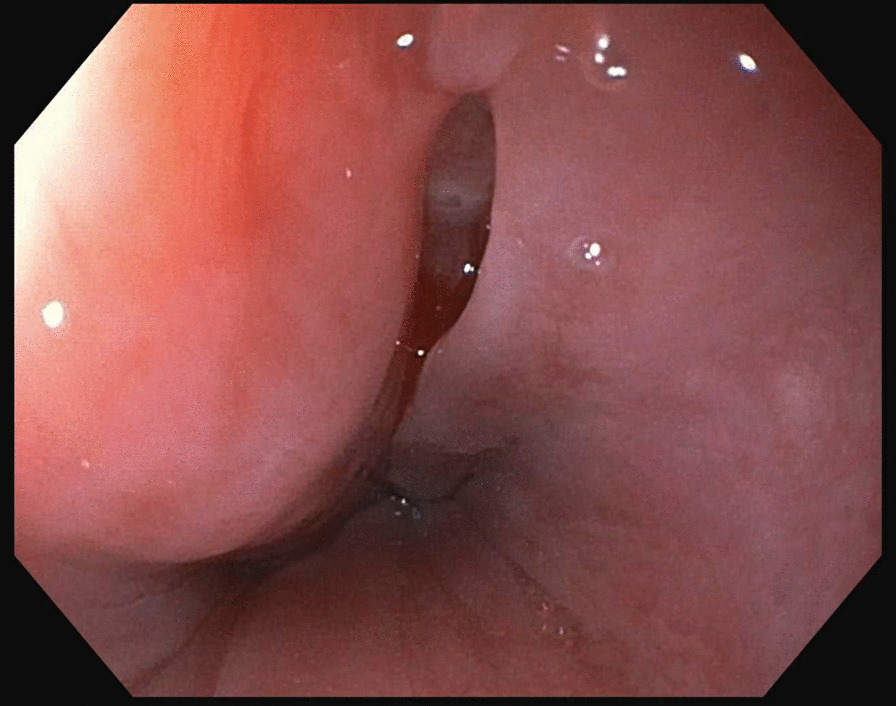
Endoscopic view of esophageal defect

**Fig. 2:**
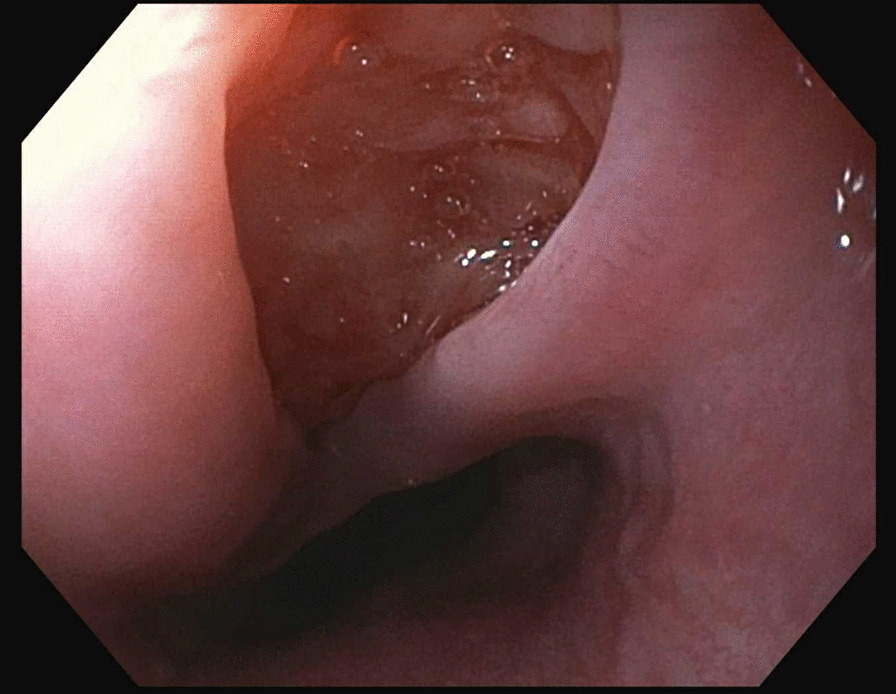
Endoscopic view of esophageal defect

**Fig. 3:**
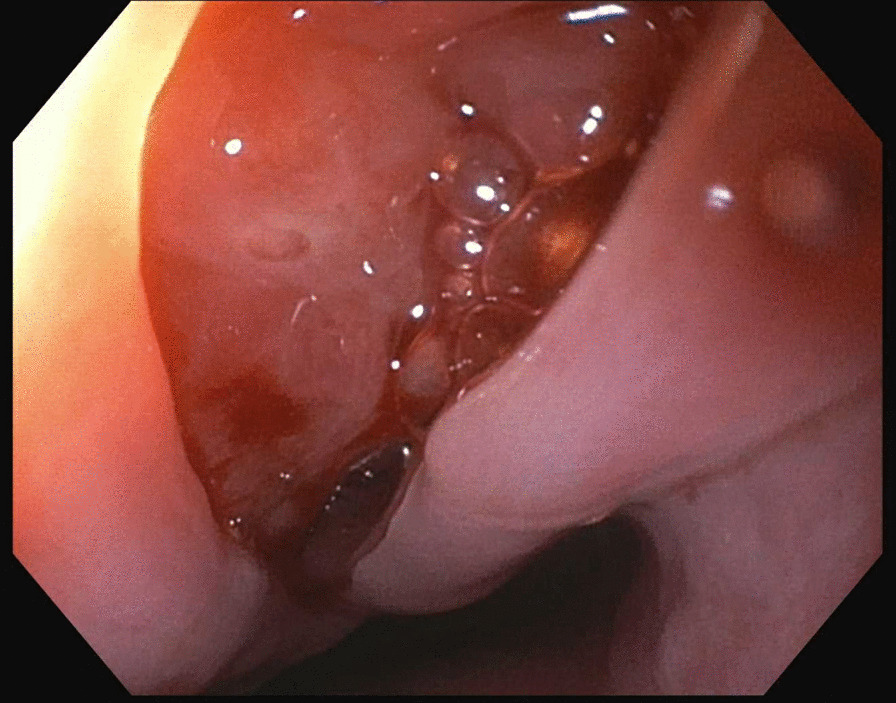
Endoscopic view of esophageal defect

A 35-year HIV-negative, Ethiopian male presented with two months of progressive dysphagia and odynophagia. He underwent an upper endoscopy that showed a mid-esophageal ulcer and was referred to our tertiary care center. He subsequently underwent an upper endoscopy that revealed a linear 2 cm full thickness defect in the middle esophagus (Figs. 1, 2, 3). Nodular tissue was visualized at the defect site, and biopsied with cold forceps.

**Fig. 4:**
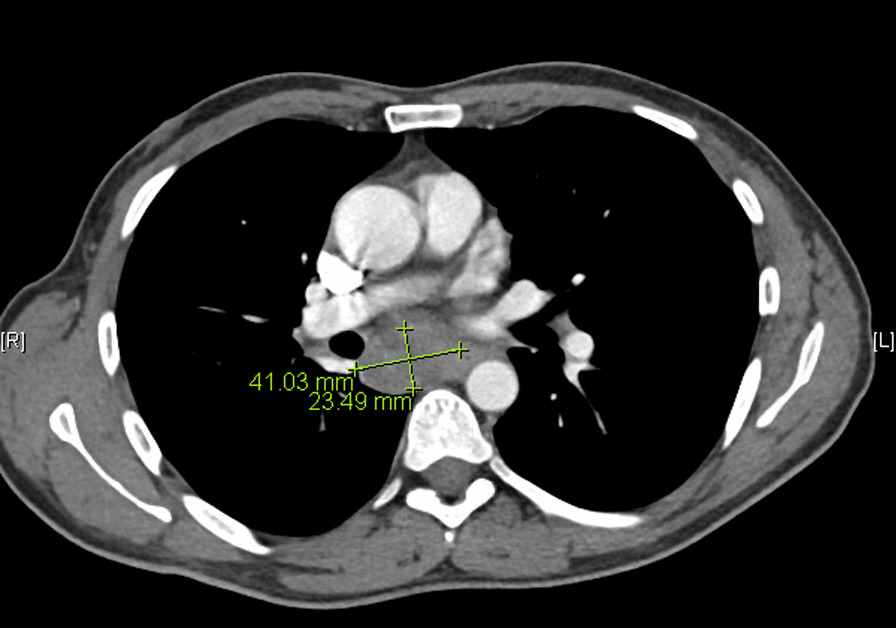
CT chest with contrast showing mediastinal mass invading esophageal wall

Histopathologic results demonstrated gastroesophageal mucosa with ulceration, granulation tissue formation, and marked reactive changes with negative Cytomegalovirus (CMV) and Herpes simplex virus (HSV) immunostains. The patient then underwent a CT chest that showed a large subcarinal soft tissue mass inseparable from the esophagus with enlarged mediastinal and hilar lymph nodes and thickening of the right suprahilar region (Fig. 4).

**Fig. 5:**
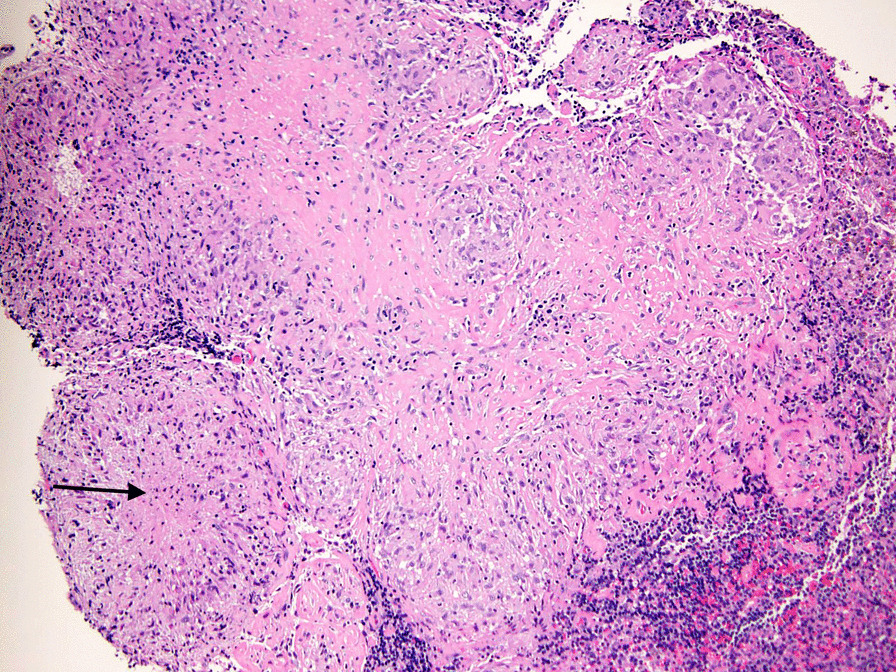
Necrotizing granulomatous inflammation involving lymph node tissue. Hematoxylin–eosin stain, ×100

Based on these findings, the patient was referred to thoracic surgery. Mediastinal dissection and mediastinoscopy was then performed with visualization and resection of several enlarged subcarinal lymph nodes. Histopathology of the resected lymph nodes were notable for necrotizing and non-necrotizing granulomas (Fig. 5), with negative fungal and AFB stains. Infectious disease was consulted based on these findings, with recommendations for empiric antifungal therapy with itraconazole and urine antigen assays for Blastomyces dermatitidis and Histoplasma capsulatum, which were negative.

Three weeks later, the AFB culture from the surgically removed lymph node resulted with *Mycobacterium TB* complex growth. Other than prior travel to Ethiopia, he denied any known TB exposures and did not have any other TB risk factors. He was then started on anti-tuberculosis treatment (ATT) with complete resolution of symptoms. At a 6-month follow-up, he reported complete resolution of his previous dysphagia and odynophagia.

## Discussion and conclusions

Gastrointestinal manifestations of TB can include involvement of the gastrointestinal tract, peritoneum, lymph nodes, and/or solid organs [4]. Lymph nodes are the most common site of involvement of EPTB. Uncommon forms of abdominal TB include involvement of the esophagus (2.8% of all cases of GI TB), stomach, duodenum, pancreas, and spleen [5].

Symptoms of Esophageal TB (ET) include dysphagia, odynophagia, chest pain, low‐grade fever, and weight loss. ET usually occurs at the middle third of the esophagus at the level of the carina [6]. Dysphagia is the most common presenting manifestation of gastrointestinal (GI) TB, although rare and occurs either due to primary involvement of the esophagus by TB or secondary to direct extension from adjacent structures [7]. Primary ET is very rare because of various protective mechanisms including the presence of stratified squamous epithelial cells covered with mucus in the esophagus [8]. In a case study that looked at 2176 patients with persistent dysphagia, 12 patients were found to have ET, and 10 of these patients had likely primary ET with no other identifiable sources of TB [9]. Primary ET should be suspected in any patient presenting with dysphagia, the typical finding of a linear ulcer, or hypertrophic growth on endoscopy [9]. Most cases of ET are secondary to direct extension from adjacent structures, such as mediastinal lymph nodes or pulmonary sites [10]. TB penetrates the mucosa and localizes in the submucosal lymphoid tissue, where it initiates an inflammatory reaction with subsequent lymphangitis, endarteritis, granuloma formation, caseation necrosis, mucosal ulceration, and scarring.

The clinical, radiological, and endoscopic features of ET are not well defined because of its rarity [11]. CT scans typically demonstrate more lesions than are visible on chest radiographs and are particularly helpful in detecting hilar or mediastinal lymphadenopathy. When there is a concern for esophageal involvement of TB, deep endoscopic biopsies are recommended to be taken from the margins of any visualized ulceration, as TB granulomas are often submucosal compared to the mucosal granulomas typically seen in Crohn’s disease [12]. Given the rarity of esophageal related TB in western populations, other differential diagnoses should be considered, including Crohn’s disease and primary esophageal malignancy [13].

Histopathology and TB‐polymerase chain reaction (PCR) are the mainstay for confirming the diagnosis of ET [14]. The presence of caseating granulomas is suggestive of tuberculosis but is not pathognomonic [15]. Studies show PCR sensitivities ranging from 74 to 100% for smear-positive specimens [16]. The sensitivity of the AFB smear and mycobacterial culture for biopsy specimens is low (< 50%) [15]. Histology shows epithelioid granulomas with Langhans cells and central caseous necrosis. Classical granulomas are seen only in 50% of cases, whereas AFB is demonstrated in < 25% of cases. Endoscopic mucosal biopsy has a sensitivity of 22% as reported by Mokoena et al. [17].

Early diagnosis and initiation of antituberculosis therapy and surgical treatment are essential to prevent morbidity and mortality [4]. Most patients respond well with ATT and patients with ET complicated with esophagotracheal and esophagomediastinal fistulas can be safely treated with ATT alone [14]. Patients with GI related TB should undergo multidisciplinary management with infectious disease consultation for the guidance of pharmacologic treatment.

The reported complications of ET include aspiration pneumonia, fatal hematemesis, esophagotracheal fistula, esophagomediastinal fistula, traction diverticula, esophageal strictures, and amyloidosis [17]. Surgical treatment is reserved for complications such as perforation, abscess formation, high-grade obstructions, and fistulas (esophageal, tracheoesophageal, and aorto-esophageal) [18].

Our patient was ultimately found to have esophageal TB secondary to mediastinal invasion into the esophageal wall from lymphadenopathy associated with TB. This is an extremely rare presentation in western populations due to diminished exposure rates and overall less immunocompromised populations compared to impoverished countries with increased TB exposure and HIV infection rates. Although TB is not as commonly seen in western populations, it should be considered on the differential for any atypical presentations of GI diseases for patients with clinical or geographic risk factors.

## Data Availability

The datasets used and/or analyzed during the current study are available from the corresponding author on reasonable request.

## Abbreviations

TB: *Mycobacterium tuberculosis*
GI: Gastrointestinal
EPTB: Extrapulmonary tuberculosis
cm: Centimeter
CT: Computed tomography
AFB: Acid Fast Bacillus
ATT: Anti-tuberculosis treatment
HIV: Human immunodeficiency virus
CMV: Cytomegalovirus
HSV: Herpes simplex virus
ET: Esophageal TB
PCR: Polymerase chain reaction
